# Omega-3-Rich Tuna Oil Derived from By-Products of the Canned Tuna Industry Enhances Memory in an Ovariectomized Rat Model of Menopause

**DOI:** 10.3390/antiox13060637

**Published:** 2024-05-24

**Authors:** Jintanaporn Wattanathorn, Wipawee Thukham-Mee

**Affiliations:** 1 Department of Physiology, Faculty of Medicine, Khon Kaen University, Khon Kaen 40002, Thailand; meewep@gmail.com; 2 Research Institute for High Human Performance and Health Promotion, Khon Kaen University, Khon Kaen 40002, Thailand

**Keywords:** tuna oil, omega 3, memory, menopause, by-product

## Abstract

To increase the value of the by-products of the canned tuna industry, the memory enhancement effect and the possible mechanisms of omega-3-rich tuna oil in bilateral ovariectomized (OVX) rats were assessed. Female rats were orally given tuna oil at doses of 140, 200, and 250 mg/kg of body weight (BW) for 28 days before OVX and for 21 days continually after OVX. Memory performance was assessed every week, whereas the parameters regarding mechanisms of action were assessed at the end of the study. All doses of tuna oil enhanced memory, docosahexaenoic acid (DHA) levels, and superoxide dismutase (SOD) and glutathione peroxidase (GPx) activities but decreased cortisol, acetylcholinesterase (AChE), malondialdehyde (MDA), and inflammatory cytokines such as tumor necrosis factor-α (TNF-α) and interleukin-6 (IL-6). Medium and high doses of tuna oil suppressed monoamine oxidase (MAO) but increased eNOS activity. A high dose of tuna oil suppressed gamma-aminotransferase (GABA-T) but increased glutamic acid decarboxylase (GAD) and sirtuin-1. A medium dose of tuna oil decreased homocysteine (Hcys) and C-reactive protein. No change in telomere or estradiol was observed in this study. Our results suggest the memory-enhancing effect of tuna oil in an OVX rat model of menopause. The main mechanisms may involve a reduction in oxidative stress, inflammation, and neurotransmitter regulation.

## 1. Introduction

Menopausal women constitute approximately 10% of the global population. It has been reported that each year, around 25 million women worldwide pass through the menopausal period [1]. The transition to menopause, that is a permanent loss of ovarian activity, leads to a decrease in estrogen and a cessation of the menstrual cycle [2]. The decrease in or the eventual cessation of estrogen has been reported to produce various menopause-related disorders including anxiety, depression, and memory impairment [2,3]. Memory deficit is recognized as an important problem in post-menopausal women [4,5,6]. Oxidative stress [7,8] and proinflammatory cytokines [9] are reported to be associated with menopause and memory deficit. Many studies also demonstrate that disturbances in neurotransmitters such as cholinergic [10], monoaminergic [11], and GABAergic [12] systems and a reduction in sirtuin-1 (sirt1), an important regulatory protein, together with a hyperhomocyteinemia [13] also play the roles in menopause associated with memory impairment [14]. In addition, it has been demonstrated that menopause is associated with endothelial dysfunction [15] and a reduction in nitric oxide [16], which in turn leads to a reduction in endothelial nitric oxide synthase (eNOS) [17] and memory deficit during menopause [18]. Several lines of evidence in this decade have revealed that the ingestion of omega-3 fatty acids increases learning, memory, cognitive well-being, and blood flow to the brain [19]. These findings raise the possibility that omega-3-enriched substances derived from tuna by-products may reduce menopause-related memory impairment by modifying the mentioned biomarkers.

The canned tuna industry has been recognized as an important export of Thailand [20]. In 2023, the canned tuna industry grew by around 15.7%, and Thailand has become the world’s second largest exporter after China [20]. Approximately 70% of tuna’s weight is discarded as a by-product during the production of canned tuna [21]. This product contains high amounts of proteins and lipids, particularly long chain polyunsaturated fatty acid (PUFA) such as omega-3 fatty acids [22], especially docosahexaenoic acid (DHA) and eicosapentaenoic acid (EPA). Omega-3 fatty acid, or “vitamin F”, has the potential to provide health benefits related to body functions and metabolism [23]. It plays an important role in regulating many cellular functions including membrane fluidity, inflammation, and the regulation of cardiovascular function, metabolism [23], and the nervous system [24]. Therefore, omega-3 fatty acid-enriched tuna oil derived from the by-products of the tuna industry has been considered as a potential functional ingredient targeting memory improvement during menopause. To date, no available scientific evidence clearly demonstrates the memory-enhancing effect of omega-3-enriched tuna oil and its possible underlying mechanisms particularly the modification effect of omega-3 tuna oil on the biomarkers mentioned earlier. Therefore, we aimed to elucidate the mentioned issue in a rat model of menopause induced with bilateral ovariectomy (OVX).

## 2. Materials and Methods

### 2.1. Tuna Oil

Tuna oil was kindly provided by Thai Union Manufacturing, Co., Ltd., Muaeng Samut Sakhon District, Samut Sakhon, Thailand. This tuna oil had been extracted from fresh tuna heads under a temperature of 40 °C. The tuna oil revealed a p-anisidine value of around 35.8. According to AOAC (2019) 990.06, the developed tuna oil contained omega-3 fatty acids of around 34.06% (33,978.39 mg/100 g) comprising 25.61% of docosahexaenoic acid (DHA) and 5.51% of eicosapentaenoic acid (EPA), whereas 5.96% of omega-6 and 12.56% of omega-9 were observed. The details of various compositions presented in tuna oil is shown in Supplementary Material S1.

### 2.2. Experimental Animals and Treatments

Virgin female Wistar rats (6–10 weeks) weighing 180–200 g were obtained from Nomura Siam International Co., Ltd., Bangkok, Thailand. They were housed in groups of 4 individuals in a metal cage under a standard condition (22 ± 2 °C in a 12-h/12-h light/dark cycle) and provided food (No. 082, produced by the Northeast Laboratory Animal Center, Khon Kaen University, Thailand, which contained protein, ≥18%; fat, ≥3%; dietary fiber, ≥24%; moisture, ≥10%; ash, ≥10%; vitamin A, 9000 international units/kg; vitamin D3, 1800 international units/kg; vitamin E, 80 international units/kg; and vitamin C, 800 mg/kg) and water ad libitum. All procedures used in this study were approved by the Institutional Animal Care and the Animal Ethics Committee of Khon Kaen University, based on the ethic of animal experimentation of national research council of Thailand (ACUC-KKU-46/2564). After 7 days of an acclimatization period, all rats were divided into 9 groups (*n* = 8/group), as described in the following.

Group I. Naïve intact control group.
Group II. DW (distilled water) + sham operation group: Rats in this group were orally administered DW with an intragastric needle for 28 days to induce the same feeding stress as that induced in the other treatment groups and were subjected to a sham operation procedure, that is, the same processes as that in bilateral ovariectomy but without actual ovariectomy being carried out. Next, they were continually administered DW with an intragastric needle once daily and provided normal diet and water for 21 days after the operation. The purpose of this group was to demonstrate the effect of a sham operation along with stress feeding, because distilled water is known to produce no effect on memory, a primary outcome in this study.
Group III. Vehicle (sunflower oil) + sham operation group: All rats were subjected to a sham operation as described in Group II, but DW administration was replaced with vehicle administration. This group could provide information about the effect of a vehicle (in this case, sunflower oil) which was used as a delivery system for tuna oil.
Group IV. Vehicle (sunflower oil) + OVX-treated group: Rats in this group were orally administered sunflower oil, a vehicle used in this study, for 28 days before the bilateral ovariectomy (OVX) and for 21 days continually after the operation. The purpose of this group was to emphasize the effect of bilateral ovariectomy (OVX).
Group V. DHA + OVX-treated group: The experimental rats were treated with DHA at a dose of 360 mg/kg of BW. This dose was selected based on a previous study that showed memory enhancement [25]. The treatment was performed for 28 days before the bilateral ovariectomy (OVX) and for 21 days continually after the operation. Based on the memory-enhancing effect of DHA mentioned earlier, we also administered DHA to OVX rats, and it also served as a positive control.
Group VI. Donepezil + OVX-treated group: Rats in this group were orally administered donepezil for 28 days before the bilateral ovariectomy (OVX) and for 21 days continually after the operation. In this group, donepezil, a standard drug used for improving memory in humans, was administered to OVX rats, and it served as a positive control group. We used this comparison with an available standard drug to find out whether it is worth moving on to clinical research, which is expensive.
Group VII. Group IX tuna oil + OVX-treated groups (doses of 140, 200, and 250 mg/kg of BW were applied): All animals were subjected to the treatment as mentioned in Group IV and V except that the experimental rats were orally fed tuna oil at the doses of 140, 200, and 250 mg/kg of BW for 28 days before the bilateral ovariectomy (OVX) and for 21 days continually after an operation. These groups were experimental groups.

**Figure 1:**
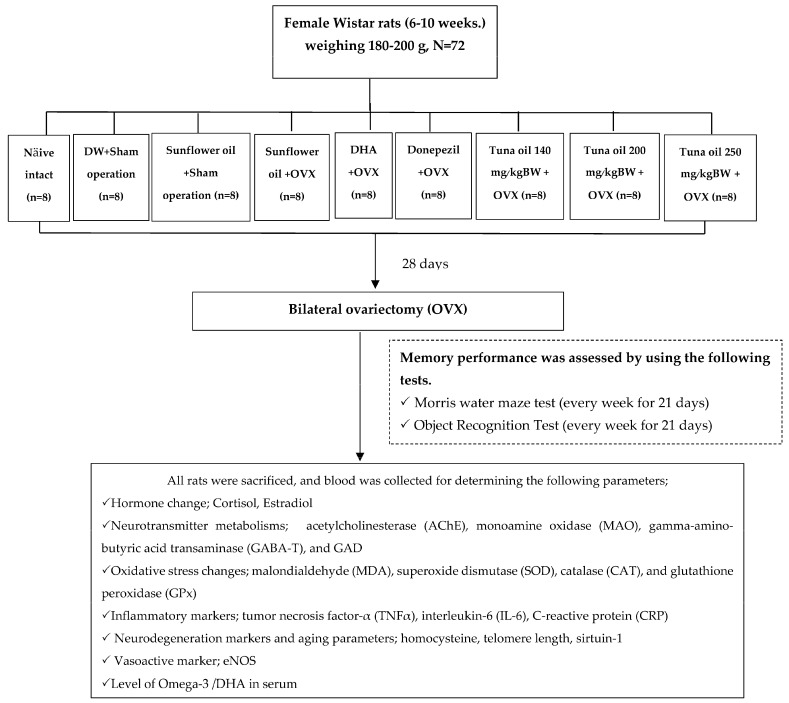
Schematic diagram of the experimental procedure.

All rats were assessed for spatial and non-spatial memory using the Morris water maze test and the object recognition test every 7 days throughout the study period. At the end of the study, the oxidative stress status including the levels of malondialdehyde (MDA) and the activities of superoxide dismutase (SOD), catalase (CAT), and glutathione peroxidase (GPx), together with the levels of inflammatory markers such as tumor necrosis factor-α (TNF-α), interleukin-6 (IL-6), and C-reactive protein (CRP) were determined. Furthermore, estradiol, blood, homocysteine (a neurodegenerative marker), telomere length, sirtuin-1, and endothelial nitric oxide synthase (eNOS) were also monitored. The alterations in neurotransmitters such as acetylcholine, monoamine, and gamma amino butyric acid (GABA) were also assessed indirectly by assessing the activities of enzymes playing important roles in the inactivation of the mentioned transmitters (AChE, MAO, GAD, and GABA-T). The serum DHA level was also measured. All experimental protocols are illustrated in Figure 1. 

### 2.3. Ovariectomy 

The surgical process was performed according to a previously reported method [26]. After anesthetization via an intraperitoneal administration of thiopental sodium (Jagsonpal Pharmaceuticals Ltd., Haryana, India) at a dose of 55 mg/kg of BW, a longitudinal incision of around 1.5 cm was made to expose the dorsolateral abdominal muscles. Following this step, the abdominal muscles and adipose tissue were pulled away to identify the ovary and the uterine horn. Next, both distal uterine horns were ligated, and ovarian tissues on both sides were removed. After the horns were returned to the abdominal cavity, the muscle and skin were sutured using Ethicon chromic sutures number 3–0 and nylon number 4 (Johnson & Johnson Ltd., Mumbai, India).

### 2.4. Memory Assessment

To assess spatial memory, the Morris water maze test was implemented. In brief, each rat was trained to memorize the relationship between its location and the location of an immersed platform in a 4-quadrant circular pool with a diameter of 147 cm and a depth of 60 cm, which contained water at a temperature of 25 ± 1 °C and was covered with non-toxic powder by using external cues. Escape latency, or the time that each rat spent locating the immersed platform, was recorded. The same test was carried out 24 h later, and the time each rat spent in the quadrant to reach the previously located immersed platform or retention memory was also recorded [27].

Non-spatial memory was also explored by using the object recognition test based on the ability to differentiate between familiar and unfamiliar objects. Briefly, each rat was given 3 min to explore 2 identical objects (A and B) in an enclosed arena (50 cm high, 80 cm long, and 60 cm wide) in a brightly lit area. Following this process, it was again exposed to the exploration test, but one of the two objects was replaced with a novel object, that is, an unfamiliar object of a different size, shape, and color. The time a rat took to explore a novel object was recorded at 0.5, 1, and 6 h after the substance administration was recorded. The discrimination index (DI) was calculated using the following equation [27].

DI = (Novel Object Exploration Time/Total Exploration Time) − (Familiar Object Exploration Time/Total Exploration Time) × 100

### 2.5. Biochemical Assessment

#### 2.5.1. Assessment of Neurotransmitter Changes

The modified spectrophotometric method of Ellman was applied to determine AChE [27]. In brief, an aliquot of brain homogenate 20 μL in volume was mixed with the reaction mixture containing 200 µL of 0.1 mM sodium phosphate and 10 μL of 0.2 M5,5′-dithio-bis-2-nitrobenzoic acid (DNTB). The mixture was incubated at room temperature for 5 min. At the end of the incubation period, 10 μL of 15 mM acetylcholine trichloride was added and subjected to a 3 min incubation period. Then, the optical density was monitored at 412 nm by using microplate reader. AChE activity was derived using the following equation.

AChE activity = (ΔA/1.36 × 10^4^) × (1/120/230)C

where ΔA is the change in absorbance per minute, and C is the concentration of the protein in the homogenate of the brain. Data were expressed as nmol/min mg protein.

Monoamine oxidase was monitored based on the oxidation of 4-aminoantipyrine, which was condensed with vanillic acid to produce a red quinoneimine dye [28]. In brief, a chromogenic solution containing 1 mM of vanillic acid (Sigma-Aldrich, Burlington, MA, USA); 500 µM of 4-aminoantipyrine (Sigma-Aldrich, USA); and peroxidase (4 U/mL) (Sigma-Aldrich, USA) dissolved in a potassium phosphate buffer (0.2 M, pH 7.6) was mixed with 50 μL of the sample solution, 50 μL of a chromium solution, and 200 μL of 500 μM of P-tyramine (Sigma-Aldrich) in a 96-well microplate. After incubation for 30 min at room temperature, an absorbance at 490 nm was monitored with a microplate reader (iMark™ Microplate Absorbance Reader, iMark™ Microplate Absorbance Reader, Bio-Rad Laboratories, Inc., Hercules, CA, USA). Data are shown as µmol/mg protein.

In the present study, GABA-T activity was assessed and used as an index for GABAergic activity. In brief, the brain homogenate was dissolved in a solution containing 0.5% triton X-100 (*v*/*v*), 5 mM of dithiothreitol, 1 mM of pyridoxal phosphate, and 10 mM of a sodium phosphate buffer (pH 7.0) in a ratio of 1:5. The mixture was subjected to a 2000 rpm centrifugation for 20 min. Following this step, an upper part of the mixture was harvested, 20 μL of which was mixed with 40 μL of the reaction solution consisting of 20 mM of GABA, 10 mM of α-ketoglutarate, and 0.05 mM of nicotinamide adenine dinucleotide (NAD) in 0.05 mM of a phosphate buffer, pH 8.0. After exposing to a 30 min incubation period, an absorbance at 340 nm was recorded with a UV–Vis spectrophotometer [29].

Glutamic acid decarboxylase (GAD) was also monitored by using an ELISA kit (My BioSource Catalog no. MBS267056) according to the brochure protocol.

#### 2.5.2. Oxidative Stress Markers

Oxidative stress markers such as malondialdehyde (MDA) levels and the activities of superoxide dismutase (SOD), catalase (CAT), and glutathione peroxidase (GPx) were assessed according to the method mentioned in the study by Srichomphu and coworkers [30].

#### 2.5.3. Inflammatory Marker Assessment

Owing to an association between inflammation and memory loss [31], we also determined the effect of tuna oil on the alteration of serum inflammatory markers such as tumor necrosis factor-α (TNF-α), interleukin-6 (IL-6), and C-reactive protein (CRP).

A rat TNF-α Elisa kit (ab108913) was used for assessing TNF-α according to the guidelines provided by the company. In brief, the sample solution (1:10), at a volume of 50 μL, was incubated in a shaking microplate for 2 h. At the end of the incubation period, the samples were washed 5 times with a 1X wash buffer at a volume of 200 μL/well. Next, 50 μL of a biotinylated TNF-α detector antibody was added. The mixture was incubated in the shaking microplate for 2 h and again washed 5 times with a 1× wash buffer at a volume of 200 μL/well. After washing, 50 μL of conjugated streptavidin-peroxidase was added to the mixture in each well, and the resulting mixture was incubated for 30 min. At the end of incubation, the mixture in each well was washed again 5 times with 200 μL of a wash buffer. Next, the mixture was mixed with 50 μL of a chromogen substrate and subjected to a 20 min incubation period. The reaction was stopped with a stop solution at a volume of 50 μL/well. Absorbance at 40 nm was recorded using an ELISA Reader.

This study determined interleulin-6 (IL-6) levels using a rat IL-6 ELISA kit (My Bioresource cat No. MBS355410). Briefly, 100 μL of the sample solution was incubated in a microplate well at 37 °C for 90 min, and 100 μL of a biotinylated antibody was added, and the mixture was incubated at 37 °C again but for 1 h. The sample in each well was washed with a wash buffer solution at a volume of 300 μL and mixed with a TMB substrate at a volume of 100 μL/well. Next, all samples were subjected to a 30 min incubation at 37 °C. Then, the reaction was stopped with 100 μL of a stop solution per well. Absorbance at 450 nm was recorded within 10 min with an ELISA Reader.

CRP was assessed by the laboratory service unit of Srinagarind Hospital, Tambon Nai Mueang, Amphoe Mueang, Khon Kaen province.

#### 2.5.4. Assessment of Endothelial Nitric Oxide Synthase (eNOS) Activity

Endothelial nitric oxide synthase (eNOS) activity in the frontal cortex was assessed by using a rat eNOS ELISA kit (My Bioresource cat No. MBS160509). In brief, aliquots of the sample at a volume of 40 μL, 10 μL of the biotinylated antibody, and 50 μL of streptavidin-HRP were mixed in a microplate and incubated at 37 °C for 1 h. Following this step, the plate was washed 5 times with 300 μL of a wash buffer solution before mixing with 50 μL of substance solution A and 50 μL of substance solution B. After mixing, it was incubated at 37 °C for 10 min, and 50 μL of a stop solution was added at the end of the incubation period. Absorbance at 450 nm was recorded.

#### 2.5.5. Measurement of Telomere Length

Rat blood DNA was extracted according to the modified method of Mumtaz et al. [32]. A fresh blood sample in an EDTA tube was diluted with a TE buffer, pH 7.8 (1:1). The mixture was subjected to a 10 min centrifugation at a speed of 12,000 rpm. Then, a pellet was resuspended in the buffer and centrifuged again until a pinkish white color of the pellet was obtained. Following this step, it was treated with sodium acetate, SDS, and proteinnase K and incubated over night at 37 °C. At the end of incubation, it was treated with a phenol/chloroform/isoamyl alcohol (25:24:1) solution and centrifuged at 12,000 rpm for 10 min. DNA in the lymphocyte layer was transferred by a pipette to a test tube and precipitated with isopropanol. Then, it was left at room temperature for 1 h and exposed to 12,000 rpm centrifugation for 10 min. The pellet containing DNA was harvested, resuspended in ethanol, and incubated for 10 min at room temperature. Next, the centrifugation was performed again, and the pellet was dried. Extracted DNA was resuspended in a TE buffer and kept at −80° C until use. DNA content was measured by using Nano drop (260/280 ≥ 1.8) according to the method specified by Joglekar and colleagues [33].

Primer sequences for the polymerase chain reaction (PCR) study were teloF (CGGTTTGTTTGGGTTTGGGTTTGGGTTTGGGTTTGGGTT), teloR (GGCTTGCCTTACCCTTACCCTTACCC TTACCCTTACCCT), 36B4F (ACTGGTCTAGGACCCGAGAAG), and 36B4R (TCAATGGTGCCTCTGGAGATT), the frequently used primers for assessing telomere length in rodents [34]. Two master mixes of the Power SYBR Green master mix were prepared for each primer. In brief, a PCR mixture containing 10 μL of the Power SYBR Green master mix, 1 μL of 2 μM of Primer-forward, 1 μL of 2 μM of Primer-reverse, 4 μL of H_2_O, and 4 μL of DNA (5 ng/uL DNA) was prepared and added into each well of the PCR plate. All samples, the negative control, and the positive control were run in duplicate. To prevent aerosol generation, an optical adhesive film was applied to seal the plate. The plate was subjected to a 3000 rpm centrifugation for 5 min at 4 °C to eliminate air bubbles and then exposed to an annealing temperature of 60 °C. The reaction was started at 95 °C for 10 min, followed by 40 cycles of the PCR process: 95 °C for 15 s and 55 °C for 60 s. Telomere length was calculated using the method explained by Wang et al. [35].

TL (kb) = 3.274 + 2.413 × (T/S)

#### 2.5.6. Sirtuin-1 Assessment

Owing to the crucial role of sirtuin on lifespan extension [36], we also determined the effect of an omega-3-enriched product on serum sirtuin-1 by using an ELISA kit (My Bioresource cat No. MBS2600246). In brief, 10 μL of the sample solution was incubated in each well of a microplate at 37 °C for 1 h. It was washed with 300 μL of a buffer solution 2 times and incubated with 100 μL of the biotinylated antibody at 37 °C for 1 h. At the end of incubation, it was washed 3 times with 300 μL of a wash buffer solution. Then, 100 μL of conjugated enzyme was added to each well and incubated at 37 °C for 30 min. Aliquots of 100 μL of a color reagent was added to stop a reaction at the end of incubation, and absorbance at 450 nm was read within 10 min by an ELISA Reader.

#### 2.5.7. Assessment of Homocysteine, Cortisol, and Estradiol

Serum levels of homocysteine, cortisol, and estradiol were evaluated by the laboratory service unit of Srinagarind Hospital, Tambon Nai Mueang, Amphoe Mueang, Khon Kaen province.

### 2.6. Measurement of Serum Levels of Docosahexaenoic Acid (DHA)

The sample preparation was prepared by mixing 300 μL of plasma and a mixture solution of hexane and isopropanol (3:2, *v*/*v*) at a volume of 1000 μL. After being mixed thoroughly, it was stored at −20 °C for 15 min before being exposed to a 10,000 rpm centrifugation at 4 °C for 10 min. The supernatant was collected and dried by nitrogen. The dried sample was dissolved in 1 mL of 80% methanol and filtered with a syringe filter at a diameter of 0.22 μm prior to an analysis.

The serum levels of DHA were analyzed using a liquid chromatography–tandem mass spectrometry (LC-MS/MS) system comprising a LCMS-8030 triple-quadrupole mass spectrometer (Shimadzu, Kyoto, Japan), operated in the ESI mode, and a Shimadzu LC-20AC series HPLC system (Shimadzu, Kyoto, Japan). The LC system was operated using the isocratic mode, separation was carried out with a C18 column with acetonitrile (solvent A) and 2 mM of ammonium acetate (solvent B) (90%:10%) as mobile phases at a flow rate of 0.3 mL/min, and the sample injection volume was 2 μL.

A triple-quadrupole mass spectrometer (Shimadzu, Kyoto, Japan), operated using an electrospray ionization (ESI) source in negative mode, was engaged in this study. The system was set up as follows: an interface temperature of 350 °C, a desolvation line of 250 °C, and a heat block temperature of 400 °C, respectively. The system was maintained at an interface voltage of 3.5 kV. Nitrogen was used as a nebulizing gas at a flow of 3 L/min. The nebulizing gas flow was maintained at 3 L/min, whereas the drying gas flow was maintained at a rate of 15 L/min. The measurement was carried out using the modified method of Serafim and colleagues [37].

### 2.7. Statistical Analysis

Data are provided as the mean ± standard error of the mean (SEM). ANOVA was applied to compare the difference between groups, and the comparison between groups was performed by Tukey’s test. The comparison at each time point, such as the memory performance, was performed by using repeated measurement ANOVA followed by post hoc Tukey’s test. A *p*-value less than 0.05 was considered significant.

## 3. Results

### 3.1. Effect of Omega-3-Rich Tuna Oil on Spatial Memory

**Figure 2:**
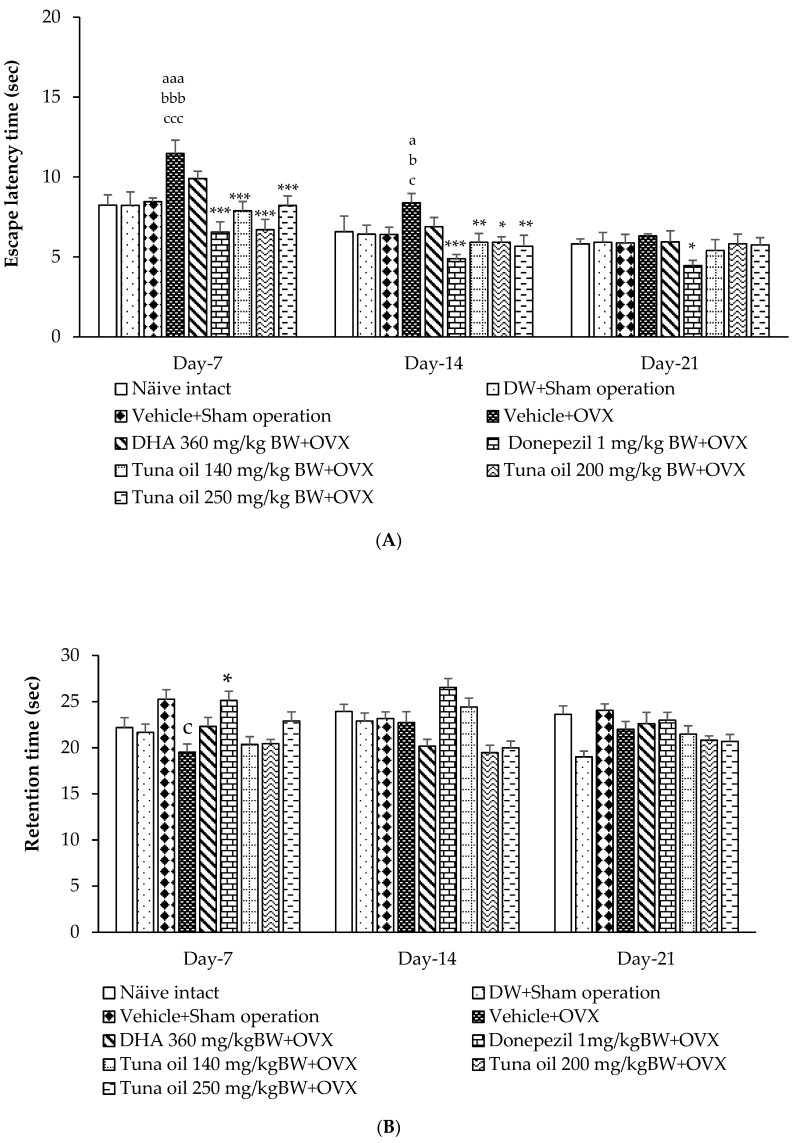
Effect of tuna oil on spatial memory assessed using the Morris water maze test. (**A**) Effect of tuna oil on escape latency. (**B**) Effect of tuna oil on retention time (*n* = 8/group) (data are expressed in mean ± SEM). ^a,aaa^ *p*-value < 0.05, 0.001, respectively, compared to the naïve control group; ^b,bbb^ *p*-value < 0.05, 0.001, respectively, compared to the DW + sham operation-treated group; ^c,ccc^ *p*-value < 0.05, 0.001, respectively, compared to the vehicle + sham operation-treated group *, **, *** *p*-value < 0.05, 0.01, and 0.001, respectively, compared to the vehicle + OVX-treated group.

The results indicate that the sham operation group that received either DW or the vehicle failed to show any significant change in escape latency when compared to the naïve control group. These results indicate that the sham operation, the DW, and the vehicle failed to exert a significant influence on escape latency. OVX rats displayed a significant decrease in escape latency on day 7 (*p*-value < 0.001, compared to the naïve control; *p*-value < 0.001, compared to the sham operation rats that received DW; *p*-value < 0.001, compared to the sham operation rats that received the vehicle) and day 14 (*p*-value < 0.05, compared to the naïve control; *p*-value < 0.05, compared to the sham operation rats that received DW; *p*-value < 0.05, compared to the sham operation rats that received the vehicle). On day 21, after OVX, the significant changes mentioned earlier disappeared. These data suggest the recovery of OVX rats, because an elevation of escape latency observed on day 7 appeared to decrease on day 14 and day 21, as shown in Figure 2A. DHA failed to produce a significant reduction in escape latency throughout the study period, whereas donepezil significantly decreased escape latency (*p*-value < 0.001, 0.001, and 0.05, compared to the vehicle + sham operation-treated group). It was found that tuna oil at doses of 140, 200, and 250 mg/kg of BW significantly decreased escape latency on day 7 (*p*-value < 0.001 all, compared to the vehicle + sham operation group) and day 14 (*p*-value < 0.01, 0.05, and 0.01, respectively, compared to the vehicle + sham operation group). However, the significant change disappeared on day 21.

The effect of tuna oil on retention memory was also explored, and the results are shown in Figure 2B. Compared to naïve intact rats, no significant change in retention time was observed in the rats subjected to the sham operation or those administered DW or the vehicle. OVX rats that received the vehicle produced a significant reduction in retention time (*p*-value < 0.05, compared to the vehicle + sham operation-treated group). However, donepezil significantly increased the retention time of OVX rats (*p*-value < 0.05, compared to the vehicle + OVX-treated group) on day 7. No other significant changes were observed. Therefore, our results suggest that omega-3-rich tuna oil did not exert an effect on retention time.

### 3.2. Effect of Omega-3-Rich Tuna Oil on Non-Spatial Memory

**Figure 3:**
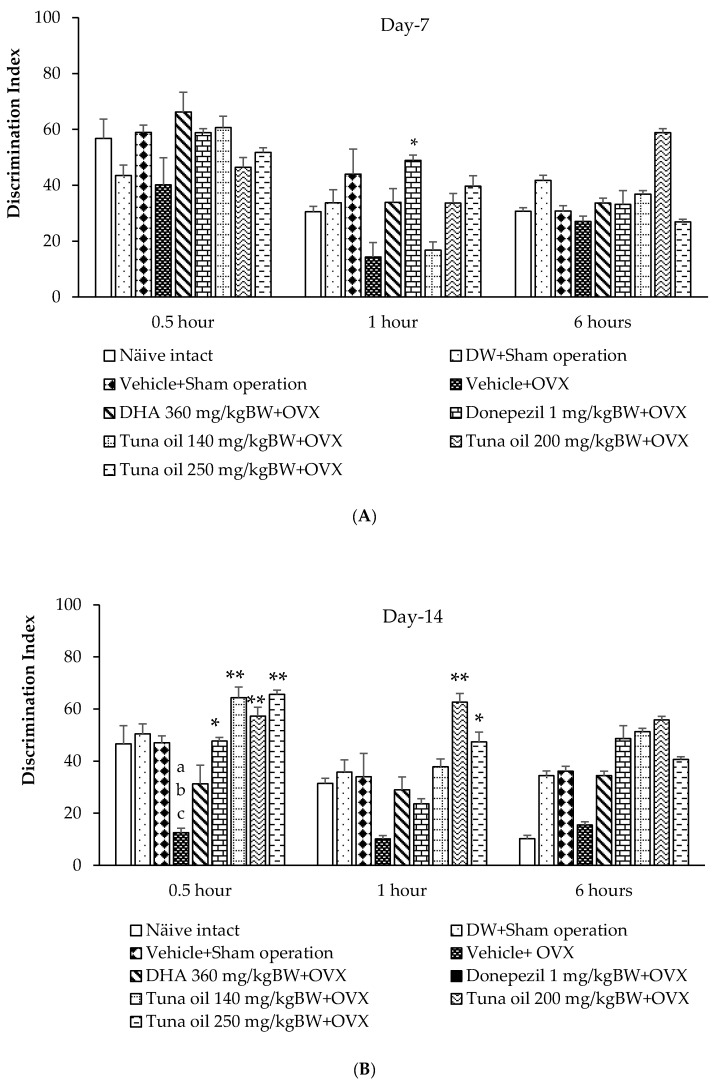

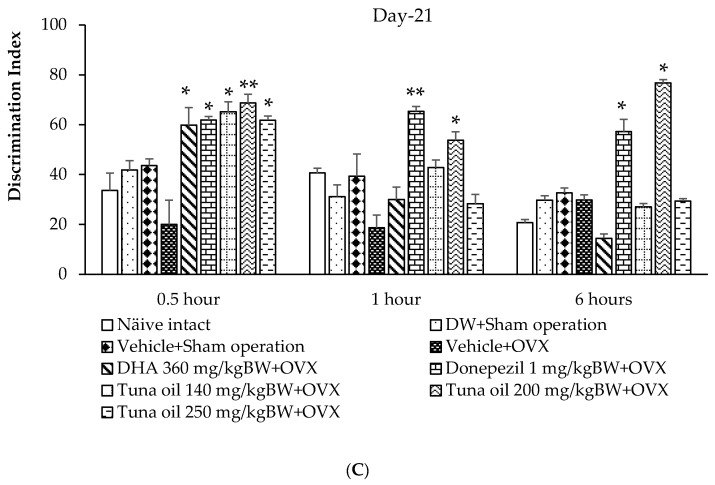
Discrimination index using an object recognition test for various treatment groups at 0.5, 1, and 6 h after substance administration. (**A**) Discrimination index at 7 days after OVX. (**B**) Discrimination index at 14 days after OVX. (**C**) Discrimination index at 21 days after OVX. Data are shown as the mean ± SEM (*n* = 8). ^a,b,c^ *p*-value < 0.05, compared to the naïve control group, the DW + sham operation group, and the vehicle + sham operation group; *, ** *p*-values < 0.05 and 0.01, respectively, compared to the vehicle + OVX-treated group.

An assessment of tuna oil influence on non-spatial memory was carried out using the novel object recognition test (NOR). Figure 3A shows that after 7 days of OVX, the only significant increase in discrimination index was observed only in OVX rats that received donepezil at 1 h after substance administration (*p*-value < 0.05, compared to the OVX + vehicle-treated group).

It was found that 14 days after OVX, the rats subjected to the sham operation that received either DW or the vehicle had failed to produce any significant change in the discrimination index throughout the assessment time points. Thirty minutes after vehicle administration, the OVX rats that received the vehicle had a significantly decreased discrimination index (*p*-value < 0.05, compared to the naïve control group; *p*-value < 0.05, compared to the DW + sham operation group; and *p*-value < 0.05, compared to the vehicle + sham operation group). Donepezil and tuna oil in all dosage ranges used in this study produced a significant improvement in this parameter in OVX rats 30 min after the administration of the substance (*p*-values < 0.05, 0.01, 0.01, and 0.01, respectively, compared to the vehicle + OVX-treated group), as shown in Figure 3B. One hour after substance administration, only OVX rats that received tuna oil treatment at the doses of both 200 and 250 mg/kg of BW showed a significant increase in the discrimination index (*p*-values < 0.01 and 0.05, respectively, compared to the vehicle + OVX-treated group). When an assessment of the novel object recognition test was performed 6 h after substance administration, no significant change in this parameter was observed.

When the treatment was prolonged to 21 days after OVX, no significant change in the discrimination index was observed in the sham operation group that received either DW or the vehicle throughout the time points of assessment. OVX rats that received the vehicle also failed to show any significant change in this parameter. DHA, donepezil, and all doses of tuna oil significantly increased the discrimination index of the OVX rats at 30 min after the administration of the substances (*p*-values < 0.05, 0.05, 0.05, 0.01, and 0.05, respectively, compared to the vehicle + OVX-treated group). However, a significant change in this parameter was observed only in OVX rats that received either donepezil or tuna oil at a dose of 200 mg/kg (*p*-values < 0.01, 0.05, 0.05, and 0.05, respectively, compared to the vehicle + OVX-treated group) at 1 and 6 h after the administration of the substance, as shown in Figure 3C.

### 3.3. Neurotransmitter Changes

**Figure 4:**
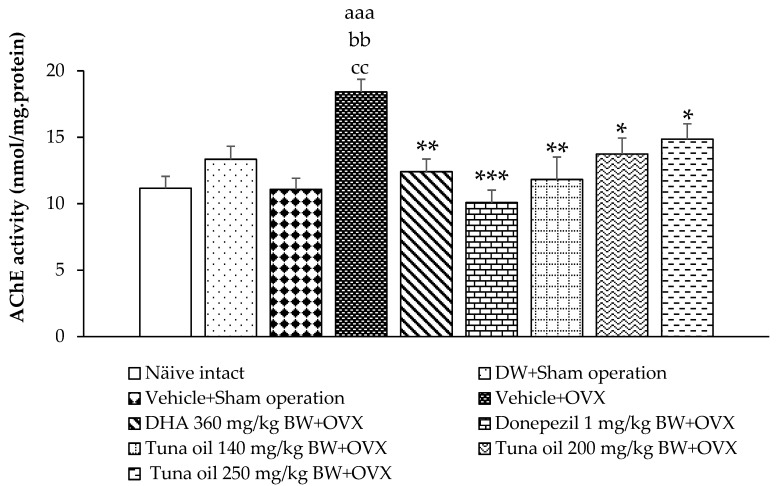
Acetylcholinesterase activity in the prefrontal cortex of various treatment groups. Data are shown as the mean ± SEM (*n* = 8/group). ^aaa^ *p*-value < 0.001, compared to the naïve control group; ^bb^ *p*-value < 0.01, compared to the DW + sham operation group; ^cc^ *p*-value < 0.01, compared to the vehicle + sham operation group; and *, **, *** *p*-values < 0.05, 0.01, and 0.001, respectively, compared to the vehicle + OVX-treated group.

**Figure 5:**
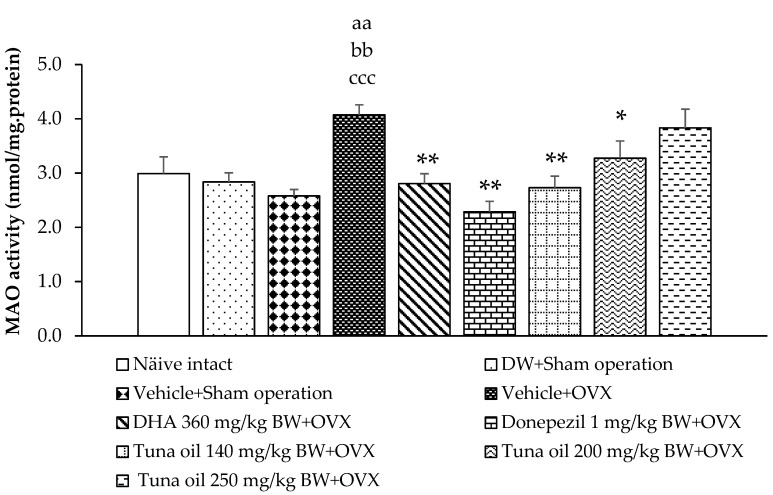
Monoamine oxidase activity in the prefrontal cortex of various treatment groups. Data are shown as the mean ± SEM (*n* = 8/group). ^aa^ *p*-value < 0.01, compared to the naïve control group; ^bb^ *p*-value < 0.01, compared to the DW + sham operation group; ^ccc^ *p*-value < 0.001, compared to the vehicle + sham operation group; and *, ** *p*-values < 0.05 and 0.01, respectively, compared to the vehicle + OVX-treated group.

**Figure 6:**
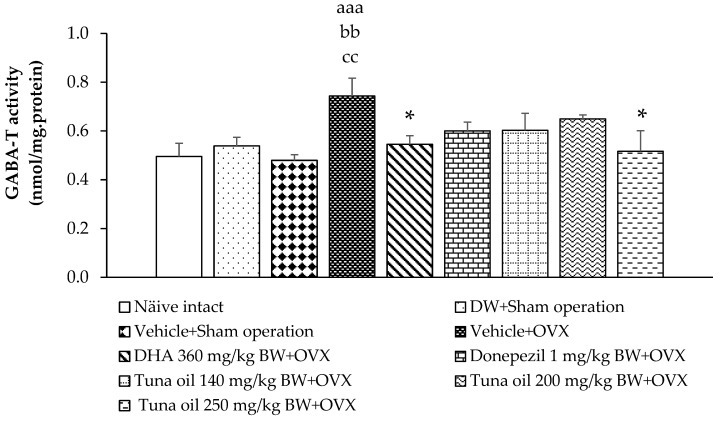
Gamma-amino-butyric acid transaminase (GABA-T) enzyme activity in the prefrontal cortex of various treatment groups. Data are shown as the mean ± SEM (*n* = 8/group). ^aaa^ *p*-value < 0.001, compared to the naïve control group; ^bb^ *p*-value < 0.01, compared to the DW + sham operation group; ^cc^ *p*-value < 0.01, compared to the vehicle + sham operation group; and * *p*-value < 0.05, compared to the vehicle + OVX-treated group.

**Figure 7:**
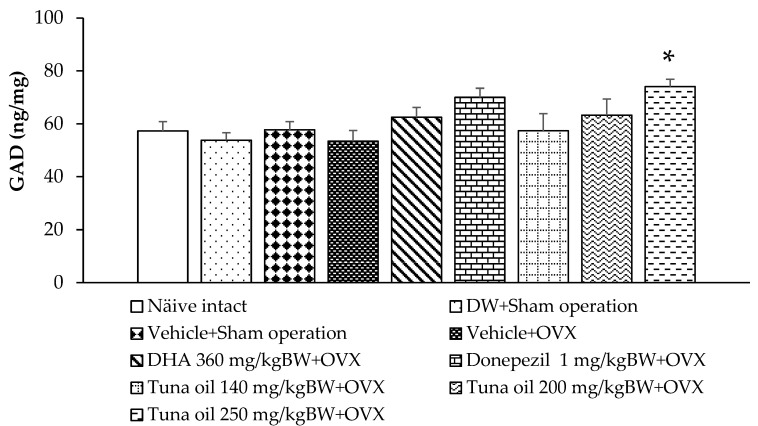
Glutamic acid decarboxylase (GAD) enzyme activity in the prefrontal cortex of various treatment groups. Data are shown as the mean ± SEM (*n* = 8/group). * *p*-value < 0.05, compared to the vehicle + OVX-treated group.

Changes in neurotransmitters influenced memory and mood. Acetylcholine, monoamine, glutamate, and gamma amino butyric acid (GABA) were indirectly determined by assessing the enzyme activities that play key roles in the turnover rates of the mentioned transmitters, including acetylcholinesterase (AChE), monoamine oxidase (MAO), glutamic acid decarboxylase (GAD), and GABA-transaminase (GABA-T). The results are demonstrated in Figure 4, Figure 5, Figure 6 and Figure 7.

Figure 4 reveals that the sham operation group that received either the DW or vehicle treatment failed to show a significant change in AChE activity in the prefrontal cortex area, whereas OVX rats that received the vehicle showed significantly enhanced AChE activity in this area (*p*-value < 0.001, compared to the naïve control group; *p*-value < 0.01, compared to the DW + sham operation group; and *p*-value < 0.01, compared to the vehicle + sham operation group). However, this elevation in AChE was improved with DHA, donepezil, and tuna oil at all doses used in this study (*p*-values < 0.01, 0.001, 0.01, 0.05, and 0.05, compared to the vehicle + OVX group).

The results also demonstrate that the sham operation group subjected to either DW or the vehicle failed to show any change in MAO activity, as shown in Figure 7. OVX rats that received tuna oil showed significantly increased brain MAO activity (*p*-value < 0.01, compared to the naïve control group; *p*-value < 0.01, compared to the DW + sham operation group; and *p*-value < 0.001, compared to the vehicle + sham operation group). However, the elevation in this enzyme was improved with DHA, donepezil, and tuna oil both at the doses of 140 and 200 mg/kg of BW (*p*-values < 0.01, 0.01, 0.01, and 0.05, respectively, compared to the vehicle + OVX), as demonstrated in Figure 5.

Compared to the naïve control group, the sham operation rats that received either DW or the vehicle also showed no significant change in GABA-T and GAD activities, as revealed in Figure 6 and Figure 7, respectively. OVX rats that received the vehicle showed increased GABA-T activity in the prefrontal cortex (*p*-value < 0.001, compared to the naïve control group; *p*-value < 0.01, compared to the DW + sham operation group; and *p*-value < 0.01, compared to the vehicle + sham operation group). DHA, donepezil, and all doses of tuna oil decreased this elevation, but a significant change was observed only in OVX rats that received either DHA or tuna oil at a dose of 250 mg/kg of BW (*p*-value < 0.05 for all, compared to the vehicle + OVX-treated group). Figure 7 shows that no significant change in GAD activity in the prefrontal cortex was observed except for a significant elevation in GAD activity in OVX rats that received tuna oil at a dose of 250 mg/kg of BW (*p*-value < 0.05, compared to the vehicle + OVX-treated group).

### 3.4. Changes in Oxidative Stress Markers

Table 1 demonstrates that no significant changes were observed in MDA, SOD, CAT, and GPx in the prefrontal cortex of the sham operation rats that received either DW or the vehicle. OVX rats that received the vehicle showed significantly increased MDA levels (*p*-value < 0.01, compared to the naïve control group; *p*-value < 0.05, compared to the DW + sham operation group; and *p*-value < 0.05, compared to the vehicle + sham operation group). However, it failed to show significant changes in SOD, CAT, and GPx in the mentioned area of OVX rats. DHA significantly decreased the MDA level (*p*-value < 0.01, compared to the vehicle + OVX-treated group) but increased the SOD activity (*p*-value < 0.05, compared to the vehicle + OVX-treated group) in the mentioned area of OVX rats. Although donepezil significantly enhanced both SOD and GPx activity (*p*-value < 0.05 for all, compared to the vehicle + OVX-treated group), it failed to produce a significant reduction in the MDA level in the prefrontal cortex of OVX rats. Interestingly, all doses of tuna oil used in this study significantly enhanced SOD (*p*-value < 0.01 for all, compared to the OVX + vehicle-treated group) and GPx (*p*-value < 0.05 for all, compared to the OVX + vehicle-treated group) activities but suppressed the MDA level (*p*-values < 0.05, 0.05, and 0.01, respectively, compared to the OVX + vehicle-treated group).

### 3.5. Inflammatory Marker Changes

**Figure 8:**
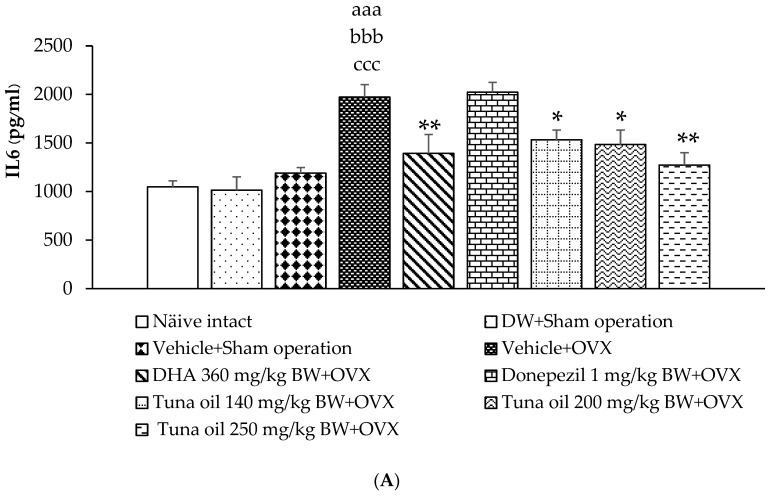

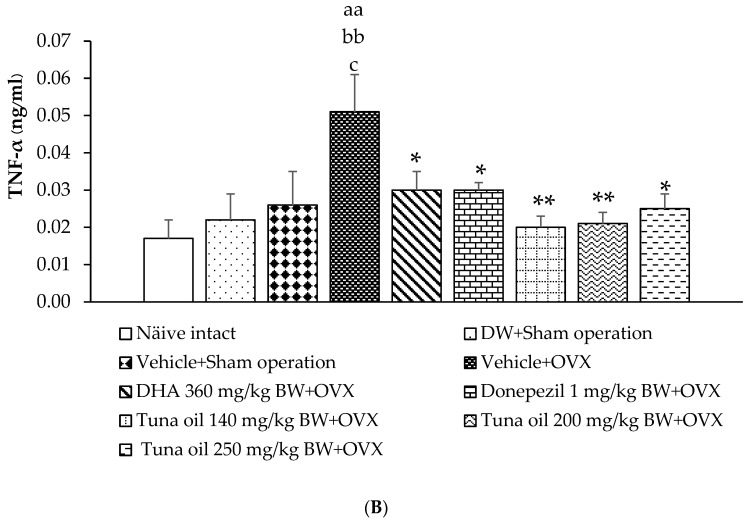

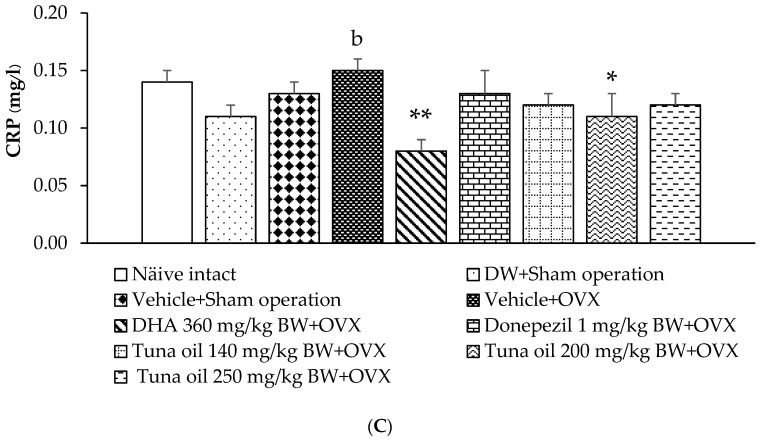
Inflammatory markers, including interleukin-6 (IL-6) (**A**), tumor necrosis factor-alpha (TNF-α) (**B**), and C-reactive protein (CRP) (**C**) in the serum of various treatment groups. Data are shown as the mean ± SEM **(***n* = 8/group). ^aa,aaa^ *p*-values < 0.01 and 0.001, respectively, compared to the naïve control group; ^b,bb,bbb^ *p*-values < 0.05, 0.01, and 0.001, respectively, compared to the DW + sham operation group; ^c,ccc^ *p*-values < 0.05 and 0.001, compared to the vehicle + sham operation groups; and *, ** *p*-values < 0.05 and 0.01, respectively, compared to the vehicle + OVX-treated group.

Figure 8A–C reveal that the sham operation procedure, DW, and the vehicle failed to show any significant changes in IL-6, TNF-α, and CRP in the serum of the experimental rats. However, OVX rats that received the vehicle showed a significantly increased IL-6 (*p*-value < 0.001, compared to the naïve control group; *p*-value < 0.001, compared to the DW + sham operation group; and *p*-value < 0.001, compared to the vehicle + sham operation group). However, this change was attenuated with DHA and all doses of tuna oil used in this study (*p*-values < 0.01, 0.05, 0.05, and 0.01, respectively, compared to the vehicle + OVX-treated group), as shown in Figure 8A. In addition, OVX increased TNF-α (*p*-value < 0.01, compared to the naïve control group; *p*-value < 0.01, compared to the DW + sham operation group; and *p*-value < 0.05, compared to the vehicle + sham operation group). The mentioned change was also mitigated with DHA, donepezil, and all doses of tuna oil (*p*-values < 0.05, 0.05, 0.01, 0.01, and 0.05, respectively, compared to the vehicle + OVX-treated group), as shown in Figure 8B. Figure 8C reveals that the level of CRP in the serum of OVX rats also increased (*p*-value < 0.05, compared to the DW + sham operation group). The medium dose of omega-3-rich tuna oil also improved this change.

### 3.6. Hormonal Changes

**Figure 9:**
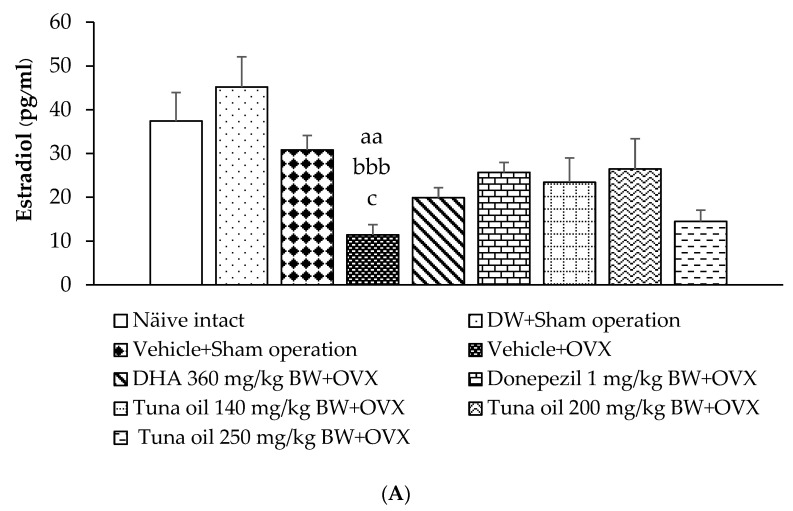

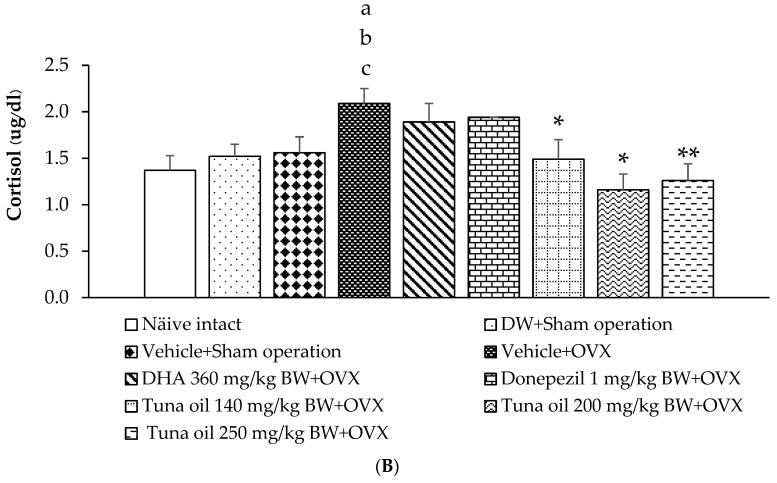
Levels of estradiol (**A**) and cortisol (**B**) in the serum of various treatment groups. Data are shown as the mean ± SEM (*n* = 8/group). ^a,aa^ *p*-values < 0.05 and 0.01, respectively, compared to the naïve control group; ^b,bbb^ *p*-values < 0.05 and 0.001, respectively, compared to the DW + sham operation group; ^c^ *p*-value < 0.05 compared to the vehicle + sham operation group; and *, ** *p*-values < 0.05 and 0.01, respectively, compared to the vehicle + OVX-treated group.

The levels of estradiol and cortisol were explored in this study, and the results are shown in Figure 9A,B. No significant changes in estradiol and cortisol were observed in the serum of the sham operation rats that received DW and those that received the vehicle. Figure 9A shows that the OVX rats that received the vehicle treatment had significantly decreased estradiol (*p*-value < 0.01, compared to the naïve control group and *p*-value < 0.01, compared to the DW + sham operation group and the vehicle + sham operation group) but increased cortisol (*p*-value < 0.05, compared to the naïve control group; *p*-value < 0.05, compared to the DW + sham operation group; and *p*-value < 0.05, compared to the sham operation + vehicle-treated group) levels in their serum, as shown in Figure 9B. Tuna oil at all doses significantly mitigated cortisol elevation in OVX rats (*p*-values < 0.05, 0.01, and 0.01, respectively, compared to the vehicle + OVX-treated group). No other significant effects were observed.

### 3.7. Changes in Various Surrogate Markers

**Figure 10:**
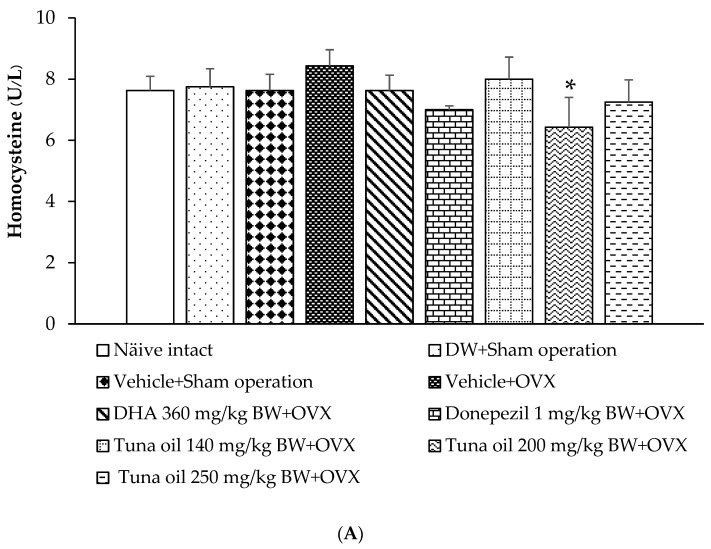

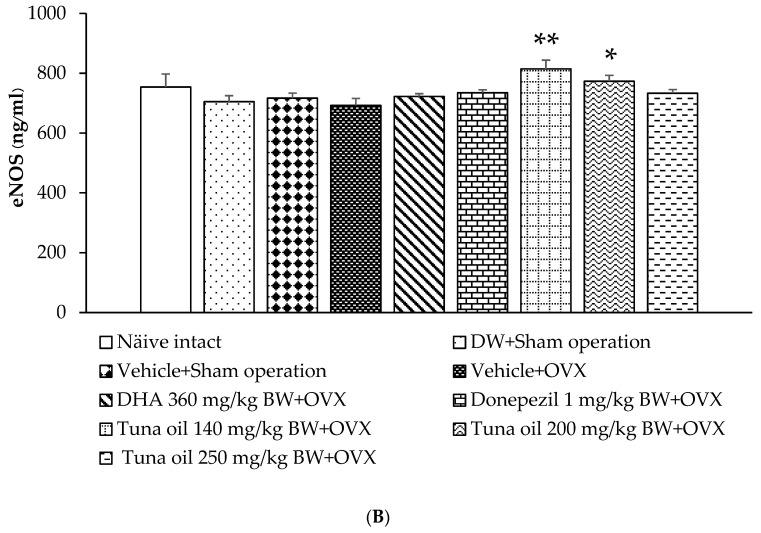

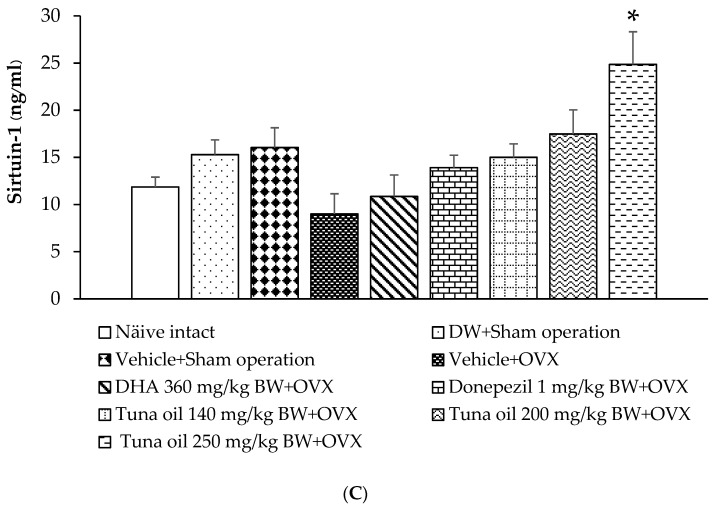

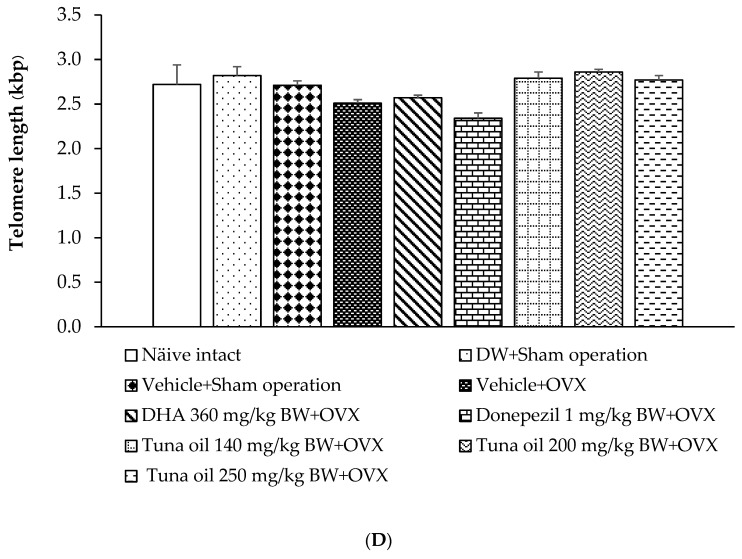
Changes in various surrogate markers, including homocysteine (Hcys) (**A**), endothelial nitric oxide synthase (eNOS) (**B**), siruin-1 (sirt-1) (**C**), and telomere length (**D**), in various treatment groups. Data are shown as the mean ± SEM (*n* = 8/group)^.^ *, ** *p*-values < 0.05 and 0.01, respectively, compared to the vehicle + OVX-treated group.

Surrogate markers, such as homocysteine, eNOS, sirtuin-1, and telomere length, were also investigated, and the results are shown in Figure 10A–D. No significant changes were found in the parameters mentioned earlier in the rats subjected to a sham operation and then administered either DW or the vehicle compared to the naïve control group. OVX rats that received the vehicle treatment also failed to show significant changes in the aforementioned parameters. Figure 10B demonstrates that tuna oil at the doses of 140 and 200 mg/kg of BW significantly increased eNOS (*p*-values < 0.01 and 0.05, respectively, compared to the OVX + vehicle-treated group), whereas tuna oil at a dose of 250 mg/kg of BW significantly increased sirtuin-1 (*p*-value < 0.05, compared to the OVX + vehicle-treated group), as shown in Figure 10C. No other changes beyond those mentioned were observed.

### 3.8. Serum DHA Levels

**Figure 11:**
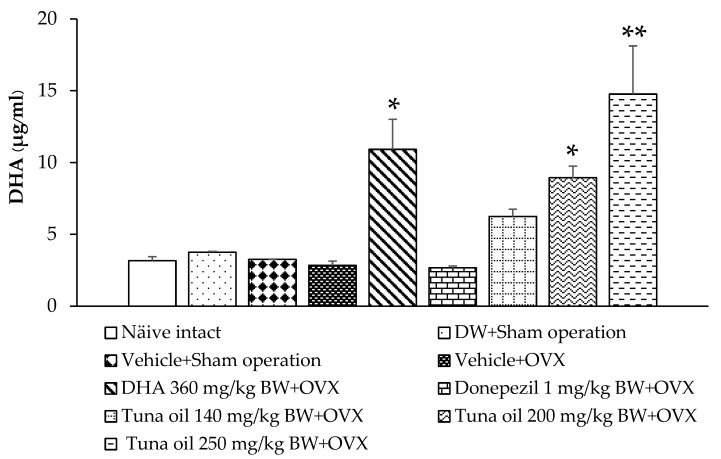
Serum levels of docosahexaenoic acid (DHA) in various treatment groups. Data are shown as the mean ± SEM (*n* = 8/group). *, ** *p*-values < 0.05 and 0.01, respectively, compared to the vehicle + OVX-treated group.

DHA, an omega-3 fatty acid, has previously been reported to improve brain function. Therefore, the DHA levels in the serum of various treatment groups were analyzed. Figure 11 shows that there was no significant change in the serum DHA levels of the sham operation rats that received DW or the vehicle compared to the naïve control group. OVX rats that received the vehicle treatment had slightly decreased serum DHA levels, but no significance was observed. DHA administration led to a significant elevation in the serum DHA levels in OVX rats (*p*-value < 0.05, compared to the vehicle + OVX-treated group). In addition, OVX rats that received tuna oil at a dosage range used in this study had increased serum DHA levels, but a significant elevation in this parameter was observed only in OVX rats that received the medium or high dose of tuna oil (*p*-values < 0.05 and 0.01, respectively, compared to the vehicle + OVX-treated group).

## 4. Discussion

This study clearly demonstrates that tuna oil derived from tuna by-products significantly improves the escape latency and discrimination index in an animal model of menopause. It suppresses AChE, MAO, GABA-T, and cortisol but enhances GAD activity in the brain. It also improves the dynamic balance of oxidative stress and regulates inflammation. It increases SOD and GPx activity, leading to a reduction in the MDA level. In addition, it decreases inflammatory indexes, such as IL-6, TNF-α, and CRP. Moreover, tuna oil at a dose of 200 mg/kg of BW also enhances MAO and eNOS activity, while a high dose of tuna oil, used in this study, or tuna oil at a dose of 250 mg/kg of BW, increases sirt-1 activity but decreases HCys. An analysis of OVX rats that received tuna oil at the dosage range used in this study reveals blood DHA levels in the range of 6.24 ± 0.51–14.76 ± 3.36 μg/mL.

Our data reveal that omega-3-rich tuna oil improves escape latency in the Morris water maze test but exerts no significant effect on retention time. The improved escape latency indicates an improved learning capability in the acquisition of spatial tasks and an improved encoding process of memory [38]. The memory process involves encoding, storage, and retrieval processes. The improved escape latency suggests an improvement in the first step of the memory or encoding process. The measurement of retention time in the probe task force of the Morris water maze test, if focused on the process of memory retention, occurs after the process of consolidation and on the retrieval process [39]. The encoding process has been identified to involve many areas. For example, item encoding involves the left hippocampus, whereas the location and time information is encoded in the right parietal region and the left fusiform region, respectively. Retrieval items also involve many areas. Item retrieval involves the right inferior frontal and parietal regions, whereas the retrieval of location and time involves the left frontal and anterior cingulate cortices, respectively [40]. Therefore, omega-3-rich-tuna oil may exert its influence in the brain areas that have a role in the encoding process rather than the areas that involve the retrieval process. Our data also demonstrate an improved discrimination index in the recognition test, which was measured with an object recognition test. This indicated an improvement in the recognition memory, because an experimental animal showed an improved ability to differentiate between the familiar and unfamiliar object [41]. This process is associated with the hippocampus and adjacent areas, such as the perirhinal, entorhinal, and parahippocampal areas [42]. Taken together, our results suggest that the hippocampus may be selectively sensitive to the effect of omega-3-rich tuna oil; therefore, we can observe an improvement in both the escape latency and discrimination index. Thus, we suggest that omega-3-rich tuna oil is a potential candidate for a memory enhancer in an OVX rat model of menopause.

Our data clearly demonstrate that OVX decreases estradiol (estrogen) but increases oxidative stress; inflammatory cytokines; and AChE, MAO, and cortisol levels. The elevation in all these parameters except cortisol corresponds with previous studies [43,44,45,46,47]. However, the effect of OVX on cortisol is still controversial. Ovariectomy in humans leads to a reduction in cortisol synthesis and secretion via a reduction in adrenocorticotrophic hormone (ACTH) stimulation [48], while an ovariectomy in animals, such as mare, increases cortisol [49], which corresponds with our results. This discrepancy may be attributed to the differences in species, health status [50], stress condition [51], and the time the blood was collected, as cortisol levels fluctuate due to circadian rhythm behavior [52].

Evidence has demonstrated that the elevation in oxidative stress and inflammation, together with a disturbance in neurotransmitter regulation, is associated with estrogen deficit [53,54,55,56,57]. However, our data have clearly demonstrated that tuna oil can improve the mentioned changes without an improvement in the estradiol level. Therefore, the effect of tuna oil may not be associated with estradiol but may be associated with a direct effect of the omega 3 in the tuna oil [58,59,60,61]. In addition to the changes mentioned earlier, the effects of tuna oil appear to depend on the administration doses. A medium dose of tuna oil improves MAO and eNOS, whereas a high dose of tuna oil improves MAO, eNOS, sirt-1, and GABA function. Data obtained from previous studies demonstrate that omega 3 can improve monoaminergic function, eNOS, and sirt-1 [62,63,64,65,66]. It also improves GABA in rat pups subjected to neurotoxicity induced with propionic acid [67] and improves serum homocysteine [68]. Therefore, we suggest that the mentioned changes may also be attributed to omega 3 in tuna oil and may not be associated with the estradiol effect, because our data fail to show a close relationship between estradiol and the observed changes.

Accumulative lines of evidence have clearly revealed that an improvement in the oxidative stress status [69,70] and inflammation [31] and a balance of neurotransmitters that play an important role in the memory process (such as acetylcholine, monoamine, and GABA [71,72,73] and eNOS [74], sirt-1 [75], cortisol [76], and Hcys [77]) are crucial for memory function. Therefore, the memory enhancement induced with tuna oil observed in this study may occur via the mechanisms just mentioned. Although this study shows the memory-enhancing effect of tuna oil derived from the by-products of the canned tuna industry in an OVX rat model of menopause, there are some limitations of this study. This model cannot represent most menopausal women, because it represents only surgical menopause, which shows abrupt and complete ovarian hormone loss. However, naturally menopausal women show a gradual ovarian follicle depletion, similar to a situation induced with 4-vinylcyclohexene-diepoxide (VCD) and followed by OVX [78]. Therefore, applications in menopausal cases may have different effects. A clinical trial study in menopausal women is essential to confirm the positive modulation effect of omega-3-rich tuna oil on memory.

This study demonstrated that tuna oil administration elevates serum DHA levels. Based on the elevation in serum DHA and the beneficial effect of DHA mentioned earlier, we suggest that memory enhancement by tuna oil may partly be attributed to DHA [79]. However, the role of EPA, which is also present in tuna oil, and its metabolites and derivatives still cannot be eliminated [79,80]. Further exploration is required to provide a better understanding of the possible active ingredients of tuna oil. No dose-dependent response was detected in this study. A possible explanation may be the lack of a simple linear relationship between the concentration of tuna oil (omega 3) and the observed parameters, because the observed parameters are under many influences. In addition, the tuna oil used in this study contains many ingredients, such as DHA and EPA, and an interaction among ingredients can also exert an influence, masking the effect of main ingredients, such as DHA.

## 5. Conclusions

This study clearly demonstrates that by-products of the canned tuna industry can be developed as omega-3-enriched functional ingredients to enhance memory and can be applied in functional food and drink, food supplement, and drug industries. Tuna oil can enhance memory in an OVX rat model of menopause via multiple pathways. The principal underlying mechanisms of all doses (140, 200, and 250 mg/kg of BW) may be associated with a reduction in the oxidative stress status because of an increase in antioxidant enzymes, such as SOD and GPx; a reduction in inflammation; and an increase in cholinergic activity. However, the increase in eNOS and monoaminergic activity may also have played a role at the medium dose, while the improvement in sirt-1 and Hcys may have played a role at the high dose used in this study. Although tuna oil shows strong potential as a memory enhancer, clinical data to confirm this study are still essential.

## Figures and Tables

**Table 1:** Oxidative stress markers, including malondialdehyde (MDA) level and the activities of superoxide dismutase (SOD), catalase (CAT), and glutathione peroxidase (GPx) enzymes in the prefrontal cortex of various treatment groups. Data are shown as the mean ± SEM (*n* = 8/group). ^aa^ *p*-value < 0.01, compared to the naïve control group; ^b^ *p*-value < 0.05, compared to the DW + sham operation group; ^c^ *p*-value < 0.05, compared to the vehicle + sham operation group; and *, ** *p*-value < 0.05 and 0.01, respectively, compared to the vehicle + OVX-treated group.

**Groups**	**Malondialdehyde Level** **(ng/mg protein)**	**Superoxide Dismutase Activity** **(U/mg protein)**	**Catalase Activity** **(U/mg protein)**	**Glutathione Peroxidase Activity** **(U/mg protein)**
Naïve intact	0.055 ± 0.004	23.481 ± 1.848	4.356 ± 0.216	2.223 ± 0.571
DW + sham operation	0.063 ± 0.003	24.541 ± 1.561	4.357 ± 0.311	2.255 ± 0.420
Vehicle + sham operation	0.064 ± 0.004	24.835 ± 1.660	3.724 ± 0.265	1.792 ± 0.623
Vehicle + OVX	0.080 ± 0.009 ^aa,b,c^	19.172 ± 1.713	4.162 ± 0.787	0.767 ± 0.180
DHA 360 mg/kg of BW + OVX	0.057 ± 0.004 **	25.683 ± 0.733 *	3.742 ± 0.308	2.968 ± 0.814
Donepezil 1 mg/kg of BW + OVX	0.071 ± 0.004	25.088 ± 0.884 *	3.931 ± 0.239	3.766 ± 0.806 *
Tuna oil 140 mg/kg of BW + OVX	0.063 ± 0.005 *	29.807 ± 3.856 **	4.888 ± 0.785	3.508 ± 0.618 *
Tuna oil 200 mg/kg of BW + OVX	0.063 ± 0.002 *	28.299 ± 2.360 **	4.948 ± 0.527	3.407 ± 0.798 *
Tuna oil 250 mg/kg of BW + OVX	0.061 ± 0.005 **	29.023 ± 2.537 **	5.037 ± 0.433	2.934 ± 0.884 *

## Data Availability

The data used to support the findings of this study are available from the corresponding author upon request.

## Supplementary Materials

The following supporting information can be downloaded at: https://www.mdpi.com/article/10.3390/antiox13060637/s1. The details of characterization methods of various ingredients and the observed concentrations are provided in supplementary material S1.
