# Supplementary material for: Omega-3-Rich Tuna Oil Derived from By-Products of the Canned Tuna Industry Enhances Memory in an Ovariectomized Rat Model of Menopause
Source: Antioxidants (Basel). 2024 May 24;13(6):637. doi: 10.3390/antiox13060637 (PMC11201088; doi:10.3390/antiox13060637)
Supplement: Supplementary file 1 [file antioxidants-13-00637-s001.zip › antioxidants-2997727-supplementary.pdf]

Client : Thai Union Group Public Company Limited
979/121 35th Floor, S.M. Tower, Phaholyothin Road,
Phaya Thai Sub-district, Phaya Thai District Bangkok 10400 Thailand

The following sample(s) was/were submitted and identified by client as:

Sample Name : Refined Tuna Oil
Sample Description : Tuna Oil
Batch : 0000288553
MFG. : 14042021

The following sample(s) was/were identified by SGS as:

SGS Sample No. : 5147577
Sample Condition : Chilled sample is contained in an aluminium bottle.

Date Received : 7-Jun-2021 Date Commenced : 9-Jun-2021

Test Items	Method	LOQ	Results	Units
Peroxide Value	AOCS Cd 8b-90	-	1.39	meq/kg
*p-Anisidine Value	AOCS Official Method Cd 18-90	-	7.27	AV
Total fatty acid	AOAC (2019) 996.06 ,GC/FID			
-Butyric acid (C4:0)		10.00	Less than 10.00	mg/100g
-Caproic acid (C6:0)		10.00	Less than 10.00	mg/100g
-Caprylic acid (C8:0)		10.00	Less than 10.00	mg/100g
-Capric acid (C10:0)		10.00	Less than 10.00	mg/100g
-Lauric acid (C12:0)		10.00	39.76	mg/100g
-Tridecanoic acid (C13:0)		10.00	44.24	mg/100g
-Myristic acid (C14:0)		10.00	3334.62	mg/100g
-Myristoleic acid (C14:1)		10.00	59.68	mg/100g
-Pentadecanoic acid (C15:0)		10.00	1062.29	mg/100g
-cis-10-Pentadecenoic acid (C15:1)		10.00	Less than 10.00	mg/100g
-Palmitic acid (C16:0)		10.00	20544.90	mg/100g
-trans-9-Hexadecenoic acid (C16:1t)		10.00	Less than 10.00	mg/100g
-Palmitoleic acid (C16:1)		10.00	4298.72	mg/100g
-Heptadecanoic acid (C17:0)		10.00	1340.62	mg/100g
-cis-10-Heptadecenoic acid (C17:1)		10.00	Less than 10.00	mg/100g

"Any holder of this document is advised that should client or third party information be supplied with respect to the goods or sample, SGS may, at its discretion, attached or indicate such information to the report but SGS makes no warranties or accepts no liable for the veracity or lack thereof of such Information."

4650625

This document is issued by the Company subject to its General Conditions of Service printed overleaf, available on request.
Attention is drawn to the limitation of liability, indemnification and jurisdiction issues defined therein. Any holder of this document is advised that information contained hereon reflects the Company's findings at the time of its intervention only and within the limits of Client's instructions, if any. The Company's sole responsibility is to its Client and this document does not exonerate parties to a transaction from exercising all their rights and obligations under the transaction documents. This document cannot be reproduced except in full, without prior written approval of the Company. Any unauthorized alteration, forgery or falsification of the content or appearance of this document is unlawful and offenders may be prosecuted to the fullest extent of the law.
Publish or advertisement of the result or this document is prohibited, unless prior written approval of the Company.
Unless otherwise stated the results shown in this test report refer only to the sample(s) received and such sample(s) are retained for 15 days only.
WARNING: The sample(s) to which the findings recorded herein (the "Findings") relate was(were) drawn and / or provided by the Client or by a third party acting at the Client's direction. The Findings constitute no warranty of the sample's representativeness of any goods and strictly relate to the sample(s). The Company accepts no liability with regard to the origin or source from which the sample(s) is/are said to be extracted.

Test Items	Method	LOQ	Results	Units
-Stearic acid (C18:0)		10.00	6301.26	mg/100g
-C18:1t (Sum3 isomer)		10.00	284.92	mg/100g
-cis-9-Oleic acid (C18:1 c)		10.00	11796.88	mg/100g
-cis-11-vacenic acid (C18:1c)		10.00	2168.37	mg/100g
-cis-12-octadecenoic acid (C18:1c)		10.00	56.44	mg/100g
-trans-9,12-Octadecadienoic acid (C18:2t)		10.00	736.17	mg/100g
-cis-9,12-Octadecadienoic acid (C18:2 c)		10.00	1244.36	mg/100g
-18:3 trans-9, trans-12, trans-15-octadecatrienoic		10.00	Less than 10.00	mg/100g
-Arachidic acid (C20:0)		10.00	412.98	mg/100g
-18:3 trans-9, trans-12, cis-15-octadecatrienoic		10.00	Less than 10.00	mg/100g
-18:3 trans-9, cis-12, trans-15-octadecatrienoic		10.00	Less than 10.00	mg/100g
-Gamma-Linolenic acid (C18:3 GLA)		10.00	123.16	mg/100g
-18:3 cis-9, trans-12, trans-15-octadecatrienoic		10.00	Less than 10.00	mg/100g
-18:3 cis-9, cis-12, trans-15-octadecatrienoic		10.00	18.05	mg/100g
-18:3 cis-9, trans-12, cis-15-octadecatrienoic		10.00	Less than 10.00	mg/100g
-18:3 trans-9, cis-12, cis-15-octadecatrienoic		10.00	22.13	mg/100g
-trans-11-Eicosenoic acid (C20:1t)		10.00	Less than 10.00	mg/100g
-alpha-Linolenic acid (C18:3 ALA)		10.00	437.79	mg/100g
-cis-11-Eicosenoic acid (C20:1)		10.00	1116.67	mg/100g
-Heneicosanoic acid (C21:0)		10.00	133.70	mg/100g
-Stearidonic acid (18:4)		10.00	642.74	mg/100g
-cis-11,14-Eicosadienoic acid (C20:2)		10.00	311.33	mg/100g
-cis-5,8,11-Eicosatrienoic acid (C20:3 n-9)		10.00	29.80	mg/100g
-Behenic acid (C22:0)		10.00	266.28	mg/100g
-cis-8,11,14-Eicosatrienoic acid (C20:3 n-6)		10.00	123.81	mg/100g
-trans-13-Docosenoic acid (C22:1t)		10.00	Less than 10.00	mg/100g

"Any holder of this document is advised that should client or third party information be supplied with respect to the goods or sample, SGS may, at its discretion, attached or indicate such information to the report but SGS makes no warranties or accepts no liable for the veracity or lack thereof of such Information."

4650625

This document is issued by the Company subject to its General Conditions of Service printed overleaf, available on request.
Attention is drawn to the limitation of liability, indemnification and jurisdiction issues defined therein. Any holder of this document is advised that information contained hereon reflects the Company's findings at the time of its intervention only and within the limits of Client's instructions, if any. The Company's sole responsibility is to its Client and this document does not exonerate parties to a transaction from exercising all their rights and obligations under the transaction documents. This document cannot be reproduced except in full, without prior written approval of the Company. Any unauthorized alteration, forgery or falsification of the content or appearance of this document is unlawful and offenders may be prosecuted to the fullest extent of the law.
Publish or advertisement of the result or this document is prohibited, unless prior written approval of the Company.
Unless otherwise stated the results shown in this test report refer only to the sample(s) received and such sample(s) are retained for 15 days only.
WARNING: The sample(s) to which the findings recorded herein (the "Findings") relate was(were) drawn and / or provided by the Client or by a third party acting at the Client's direction. The Findings constitute no warranty of the sample's representativeness of any goods and strictly relate to the sample(s). The Company accepts no liability with regard to the origin or source from which the sample(s) is/are said to be extracted.

Test Items	Method	LOQ	Results	Units
-cis-11,14,17-Eicosatrienoic acid (C20:3 n-3)		10.00	301.83	mg/100g
-cis-5,8,11,14-Eicosatetraenoic acid (C20:4 ARA)		10.00	2026.97	mg/100g
-Erucic acid (C22:1)		10.00	129.85	mg/100g
-Tricosanoic acid (C23:0)		10.00	112.90	mg/100g
-cis 8,11,14,17 Eicosatetraenoic acid C20:4(n-3)		10.00	537.68	mg/100g
-cis-13,16-Docosadienoic acid (C22:2)		10.00	43.96	mg/100g
-cis-5,8,11,14,17-Eicosapentaenoic acid (C20:5 EPA)		10.00	5524.14	mg/100g
-Lignoceric acid (C24:0)		10.00	227.44	mg/100g
-cis-13,16,19-Docosatrienoic acid (C22:3)		10.00	21.58	mg/100g
-Nervonic acid (C24:1)		10.00	537.63	mg/100g
-Adrenic acid (C22:4)		10.00	358.24	mg/100g
-Docosapentaenoic acid (C22:5)		10.00	2045.84	mg/100g
-cis-7,10,13,16,19-Docosapentaenoic acid (C22:5 DPA)		10.00	1793.16	mg/100g
-cis-4,7,10,13,16,19-Docosahexaenoic acid (C22:6 DHA)		10.00	24719.47	mg/100g
Free Fatty Acids as Oleic acid	AOCS Ab 5-49 and Ca 5a-40	-	0.04	%
Trans-fatty acid	AOAC (2019) 996.06 GC/FID			
-trans-fatty acid		10.00	1061.27	mg/100g
-trans-9-Hexadecenoic acid (C16:1)		10.00	Less than 10.00	mg/100g
-trans-6-Petroselaidic acid (C18:1)		10.00	15.32	mg/100g
-trans-9-Elaidic acid (C18:1)		10.00	167.02	mg/100g
-trans-11-Vaccenic acid (C18:1)		10.00	102.58	mg/100g
-sum isomer trans--Octadecadienoic acid (C18:2)		10.00	736.17	mg/100g
-trans-9,trans-12,trans-15-octadecatrienoic (C18:3)		10.00	Less than 10.00	mg/100g

"Any holder of this document is advised that should client or third party information be supplied with respect to the goods or sample, SGS may, at its discretion, attached or indicate such information to the report but SGS makes no warranties or accepts no liable for the veracity or lack thereof of such Information."

4650625

This document is issued by the Company subject to its General Conditions of Service printed overleaf, available on request.
Attention is drawn to the limitation of liability, indemnification and jurisdiction issues defined therein. Any holder of this document is advised that information contained hereon reflects the Company's findings at the time of its intervention only and within the limits of Client's instructions, if any. The Company's sole responsibility is to its Client and this document does not exonerate parties to a transaction from exercising all their rights and obligations under the transaction documents. This document cannot be reproduced except in full, without prior written approval of the Company. Any unauthorized alteration, forgery or falsification of the content or appearance of this document is unlawful and offenders may be prosecuted to the fullest extent of the law.
Publish or advertisement of the result or this document is prohibited, unless prior written approval of the Company.
Unless otherwise stated the results shown in this test report refer only to the sample(s) received and such sample(s) are retained for 15 days only.
WARNING: The sample(s) to which the findings recorded herein (the "Findings") relate was(were) drawn and / or provided by the Client or by a third party acting at the Client's direction. The Findings constitute no warranty of the sample's representativeness of any goods and strictly relate to the sample(s). The Company accepts no liability with regard to the origin or source from which the sample(s) is/are said to be extracted.

Test Items	Method	LOQ	Results	Units
-trans-9, trans-12, cis-15-octadecatrienoic (C18:3)		10.00	Less than 10.00	mg/100g
-trans-9, cis-12, trans-15-octadecatrienoic (C18:3)		10.00	Less than 10.00	mg/100g
-cis-9, trans-12, trans-15-octadecatrienoic (C18:3)		10.00	Less than 10.00	mg/100g
-cis-9, cis-12, trans-15-octadecatrienoic (C18:3)		10.00	18.05	mg/100g
-cis-9, trans-12, cis-15-octadecatrienoic (C18:3)		10.00	Less than 10.00	mg/100g
-trans-9, cis-12, cis-15-octadecatrienoic (C18:3)		10.00	22.13	mg/100g
-trans-11-Eicosenoic acid (C20:1)		10.00	Less than 10.00	mg/100g
-trans-13-Docosenoic acid (C22:1)		10.00	Less than 10.00	mg/100g
Acid Value	In-house method SOP No. LBAG-00127 based on AOCS Cd 3d-63 and Ab 5-49	-	0.07	mg KOH/g
Saturated Fat	AOAC (2019) 996.06,GC/FID			
-Saturated Fat		10.00	33820.99	mg/100g
-Butyric acid (C4:0)		10.00	Less than 10.00	mg/100g
-Caproic acid (C6:0)		10.00	Less than 10.00	mg/100g
-Caprylic acid (C8:0)		10.00	Less than 10.00	mg/100g
-Capric acid (C10:0)		10.00	Less than 10.00	mg/100g
-Lauric acid (C12:0)		10.00	39.76	mg/100g
-Tridecanoic acid (C13:0)		10.00	44.24	mg/100g
-Myristic acid (C14:0)		10.00	3334.62	mg/100g
-Pentadecanoic acid (C15:0)		10.00	1062.29	mg/100g
-Plamitic acid (C16:0)		10.00	20544.90	mg/100g
-Heptadecanoic acid (C17:0)		10.00	1340.62	mg/100g
-Stearic acid (C18:0)		10.00	6301.26	mg/100g
-Arachidic acid (C20:0)		10.00	412.98	mg/100g

"Any holder of this document is advised that should client or third party information be supplied with respect to the goods or sample, SGS may, at its discretion, attached or indicate such information to the report but SGS makes no warranties or accepts no liable for the veracity or lack thereof of such Information."

4650625

This document is issued by the Company subject to its General Conditions of Service printed overleaf, available on request.
Attention is drawn to the limitation of liability, indemnification and jurisdiction issues defined therein. Any holder of this document is advised that information contained hereon reflects the Company's findings at the time of its intervention only and within the limits of Client's instructions, if any. The Company's sole responsibility is to its Client and this document does not exonerate parties to a transaction from exercising all their rights and obligations under the transaction documents. This document cannot be reproduced except in full, without prior written approval of the Company. Any unauthorized alteration, forgery or falsification of the content or appearance of this document is unlawful and offenders may be prosecuted to the fullest extent of the law.
Publish or advertisement of the result or this document is prohibited, unless prior written approval of the Company.
Unless otherwise stated the results shown in this test report refer only to the sample(s) received and such sample(s) are retained for 15 days only.
WARNING: The sample(s) to which the findings recorded herein (the "Findings") relate was(were) drawn and / or provided by the Client or by a third party acting at the Client's direction. The Findings constitute no warranty of the sample's representativeness of any goods and strictly relate to the sample(s). The Company accepts no liability with regard to the origin or source from which the sample(s) is/are said to be extracted.

Test Items	Method	LOQ	Results	Units
-Heneicosanoic acid (C21:0)		10.00	133.70	mg/100g
-Behenic acid (C22:0)		10.00	266.28	mg/100g
-Tricosanoic acid (C23:0)		10.00	112.90	mg/100g
-Lignoceric acid (C24:0)		10.00	227.44	mg/100g
*Density @25 deg C	Density meter	-	0.9235	g/cm3
Monounsaturated Fat	AOAC (2019) 996.06 GC/FID			
-Monounsaturated Fat		10.00	20164.24	mg/100g
-Myristoleic acid (C14:1)		10.00	59.68	mg/100g
-cis-10-Pentadecenoic acid (C15:1)		10.00	Less than 10.00	mg/100g
-Palmitoleic acid (C16:1)		10.00	4298.72	mg/100g
-cis-10-Heptadecenoic acid (C17:1)		10.00	Less than 10.00	mg/100g
-cis-9-Oleic acid (C18:1)		10.00	11796.88	mg/100g
-cis-11-vacenic acid (C18:1c)		10.00	2168.37	mg/100g
-cis-12-octadecenoic acid (C18:1c)		10.00	56.44	mg/100g
-cis-11-Eicosenoic acid (C20:1)		10.00	1116.67	mg/100g
-Erucic acid (C22:1)		10.00	129.85	mg/100g
-Nervonic acid (C24:1)		10.00	537.63	mg/100g
Polyunsaturated Fat	AOAC (2019) 996.06 GC/FID			
-Polyunsaturated Fat		10.00	40285.86	mg/100g
-cis-9,12-Octadecadienoic acid (C18:2)		10.00	1244.36	mg/100g
-Gamma-Linolenic acid (C18:3, GLA)		10.00	123.16	mg/100g
-alpha-Linolenic acid (C18:3,ALA)		10.00	437.79	mg/100g
-Stearidonic acid (C18:4)		10.00	642.74	mg/100g
-cis-11,14-Eicosadienoic acid (C20:2)		10.00	311.33	mg/100g
-cis-11,14,17-Eicosatrienoic acid (C20:3)		10.00	301.83	mg/100g
-cis-8,11,14-Eicosatrienoic acid (C20:3)		10.00	123.81	mg/100g
-cis-5,8,11,14-Eicosatetraenoic acid (C20:4)		10.00	2026.97	mg/100g

"Any holder of this document is advised that should client or third party information be supplied with respect to the goods or sample, SGS may, at its discretion, attached or indicate such information to the report but SGS makes no warranties or accepts no liable for the veracity or lack thereof of such Information."

4650625

This document is issued by the Company subject to its General Conditions of Service printed overleaf, available on request.
Attention is drawn to the limitation of liability, indemnification and jurisdiction issues defined therein. Any holder of this document is advised that information contained hereon reflects the Company's findings at the time of its intervention only and within the limits of Client's instructions, if any. The Company's sole responsibility is to its Client and this document does not exonerate parties to a transaction from exercising all their rights and obligations under the transaction documents. This document cannot be reproduced except in full, without prior written approval of the Company. Any unauthorized alteration, forgery or falsification of the content or appearance of this document is unlawful and offenders may be prosecuted to the fullest extent of the law.
Publish or advertisement of the result or this document is prohibited, unless prior written approval of the Company.
Unless otherwise stated the results shown in this test report refer only to the sample(s) received and such sample(s) are retained for 15 days only.
WARNING: The sample(s) to which the findings recorded herein (the "Findings") relate was(were) drawn and / or provided by the Client or by a third party acting at the Client's direction. The Findings constitute no warranty of the sample's representativeness of any goods and strictly relate to the sample(s). The Company accepts no liability with regard to the origin or source from which the sample(s) is/are said to be extracted.

Test Items	Method	LOQ	Results	Units
-cis-13,16-Docosadienoic acid (C22:2)		10.00	43.96	mg/100g
-cis-5,8,11,14,17-Eicosapenta enoic acid (C20:5)		10.00	5524.14	mg/100g
-cis-13,16,19-Docosatrienoic acid (C22:3)		10.00	21.58	mg/100g
-Adrenic acid (C22:4)		10.00	358.24	mg/100g
-Docosapentaenoic acid (C22:5)		10.00	2045.84	mg/100g
-cis-7,10,13,16,19-Docosapen taenoic acid (C22:5,DPA)		10.00	1793.16	mg/100g
-cis-4,7,10,13,16,19-Docosah exaenoic acid (C22:6)		10.00	24719.47	mg/100g
-cis 8,11,14,17 Eicosatetraenoic acid (C20:4 n-3)		10.00	537.68	mg/100g
-cis-5,8,11-Eicosatrienoic acid (C20:3 n-9)		10.00	29.80	mg/100g
Omega 3	AOAC (2019) 996.06			
-Total Omega 3		10.00	33978.39	mg/100g
-ALA (C18:3n-3)		10.00	437.79	mg/100g
-Stearidonic acid (C18:4n-3)		10.00	642.74	mg/100g
-cis-11,14,17-Eicosatrienoic acid (C20:3n-3)		10.00	301.83	mg/100g
-EPA (C20:5n-3)		10.00	5524.14	mg/100g
-cis-13,16,19-Docosatrienoic acid (C22:3n-3)		10.00	21.58	mg/100g
-DPA (C22:5n-3)		10.00	1793.16	mg/100g
-DHA (C22:6n-3)		10.00	24719.47	mg/100g
-cis-9,11,14,17-eicosatetraeno ate acid (C20:4n-3)		10.00	537.68	mg/100g
Omega 6	AOAC (2019) 996.06			
-Total Omega 6		10.00	6277.67	mg/100g
-Linoleic acid (C18:2)		10.00	1244.36	mg/100g
-Gamma-Linolenic acid (GLA, C18:3)		10.00	123.16	mg/100g
-Eicosadienoic acid (C20:2)		10.00	311.33	mg/100g
-Homoogamma-Linolenic acid (C20:3)		10.00	123.81	mg/100g

"Any holder of this document is advised that should client or third party information be supplied with respect to the goods or sample, SGS may, at its discretion, attached or indicate such information to the report but SGS makes no warranties or accepts no liable for the veracity or lack thereof of such Information."

4650625

This document is issued by the Company subject to its General Conditions of Service printed overleaf, available on request.
Attention is drawn to the limitation of liability, indemnification and jurisdiction issues defined therein. Any holder of this document is advised that information contained hereon reflects the Company's findings at the time of its intervention only and within the limits of Client's instructions, if any. The Company's sole responsibility is to its Client and this document does not exonerate parties to a transaction from exercising all their rights and obligations under the transaction documents. This document cannot be reproduced except in full, without prior written approval of the Company. Any unauthorized alteration, forgery or falsification of the content or appearance of this document is unlawful and offenders may be prosecuted to the fullest extent of the law.
Publish or advertisement of the result or this document is prohibited, unless prior written approval of the Company.
Unless otherwise stated the results shown in this test report refer only to the sample(s) received and such sample(s) are retained for 15 days only.
WARNING: The sample(s) to which the findings recorded herein (the "Findings") relate was(were) drawn and / or provided by the Client or by a third party acting at the Client's direction. The Findings constitute no warranty of the sample's representativeness of any goods and strictly relate to the sample(s). The Company accepts no liability with regard to the origin or source from which the sample(s) is/are said to be extracted.

Test Items	Method	LOQ	Results	Units
-Arachidonic acid (ARA, C20:4)		10.00	2026.97	mg/100g
-Docosadienoic acid (C22:2)		10.00	43.96	mg/100g
-Adrenic acid (C22:4)		10.00	358.24	mg/100g
-Docosapentaenoic acid (C22:5)		10.00	2045.84	mg/100g
Omega 9	AOAC (2019) 996.06			
-Total Omega 9		10.00	13610.83	mg/100g
-Oleic acid (C18:1)		10.00	11796.88	mg/100g
-Eicosenoic acid (C20:1)		10.00	1116.67	mg/100g
-Erucic acid (C22:1)		10.00	129.85	mg/100g
-Nervonic acid (C24:1)		10.00	537.63	mg/100g
-cis-5,8,11- Eicosatrienoic acid (C20:3n-9)		10.00	29.80	mg/100g

Remark : 1. LOQ = "Limit of Quantitation"
2. Less than = Lower than LOQ "Limit of Quantitation"

Test(s) marked * on this Report are not included in the BLQS DMSc Accreditation Scope.

Signed for and on behalf of SGS (Thailand) Limited

Napaporn Thongthang
Acting - Multi Lab Manager

** End of Report **

"Any holder of this document is advised that should client or third party information be supplied with respect to the goods or sample, SGS may, at its discretion, attached or indicate such information to the report but SGS makes no warranties or accepts no liable for the veracity or lack thereof of such Information."

This document is issued by the Company subject to its General Conditions of Service printed overleaf, available on request.
Attention is drawn to the limitation of liability, indemnification and jurisdiction issues defined therein. Any holder of this document is advised that information contained hereon reflects the Company's findings at the time of its intervention only and within the limits of Client's instructions, if any. The Company's sole responsibility is to its Client and this document does not exonerate parties to a transaction from exercising all their rights and obligations under the transaction documents. This document cannot be reproduced except in full, without prior written approval of the Company. Any unauthorized alteration, forgery or falsification of the content or appearance of this document is unlawful and offenders may be prosecuted to the fullest extent of the law.
Publish or advertisement of the result or this document is prohibited, unless prior written approval of the Company.
Unless otherwise stated the results shown in this test report refer only to the sample(s) received and such sample(s) are retained for 15 days only.
WARNING: The sample(s) to which the findings recorded herein (the "Findings") relate was(were) drawn and / or provided by the Client or by a third party acting at the Client's direction. The Findings constitute no warranty of the sample's representativeness of any goods and strictly relate to the sample(s). The Company accepts no liability with regard to the origin or source from which the sample(s) is/are said to be extracted.

4650625
